# Acupuncture for lateral epicondylitis: A prisma-compliant protocol for a systematic review and meta-analysis of randomized controlled trials

**DOI:** 10.1097/MD.0000000000022008

**Published:** 2020-09-11

**Authors:** Ha-Na Kim, Bonhyuk Goo, Sang-Soo Nam

**Affiliations:** a Department of Clinical Korean Medicine, Graduate School, Kyung Hee University, Kyungheedae-ro, Dongdaemun-gu; b Department of Acupuncture & Moxibustion, Kyung Hee University Hospital, Gangdong, Dongnam-ro, Gangdong-gu; c Department of Acupuncture & Moxibustion, College of Korean Medicine, Kyung Hee University, Kyungheedae-ro, Dongdaemun-gu, Seoul, Republic of Korea.

**Keywords:** acupuncture, lateral epicondylitis, randomized controlled trials, systematic review, tennis elbow

## Abstract

**Background:**

Acupuncture has been widely used for relieving pain associated with musculoskeletal disorders, such as lateral epicondylitis. Although the effect of acupuncture on pain has been demonstrated in previous reviews, it is still under debate. This study is aimed at evaluating the efficacy of acupuncture to treat lateral epicondylitis and establishing the evidence systematically.

**Methods:**

Nine databases will be searched from their inception to May 2020 without language or publication status restrictions, including 3 English databases (MEDLINE, Embase, the Cochrane Central Register of Controlled Trials), 5 Korean databases (Korean Medical Database, KoreaMed, Korean Studies Information Service System, Research Information Service System, Oriental Medicine Advanced Searching Integrated System), and 1 Chinese database (China Knowledge Network Database). Only randomized controlled trials will be included. Pain intensity will be considered as the primary outcome. Secondary outcomes will include the grip strength, total effective rate, and adverse events. Two independent researchers will perform the study selection, data extraction, and quality assessment. The methodological quality of the identified studies will be assessed using the Cochrane Collaboration's risk-of-bias tool. In the meta-analysis, continuous data will be expressed as mean and 95% confidence interval, and dichotomous data will be expressed as risk ratio and 95% confidence interval.

**Results:**

The results of this study will be submitted to a peer-reviewed journal for publication.

**Conclusion:**

The results of this study would provide the evidence of whether acupuncture is effective for lateral epicondylitis.

**Registration number:**

PROSPERO CRD42020186824

## 1 Introduction

Lateral epicondylitis, or “tennis elbow”, is a common cause of elbow joint pain. It is a common clinical condition estimated to affect 1% to 3% of adults each year, and the highest incidence is among individuals aged 40 to 49 years.^[1]^ LE is commonly caused by repetitive overuse of the wrist extensor tendon, particularly the extensor carpi radialis brevis;^[2]^ therefore, those who use their hands and forearms occupationally or for sports excessively show a tendency to develop lateral epicondylitis.^[3]^ Patients report tenderness on palpation of the lateral humeral epicondyle, where the wrist extensor tendon originates. Pain in the same area is aggravated by the resisted middle finger and wrist extension, and if worsened, pain radiates down the forearm. Functional disability, such as reduction in grip strength, may occur secondary to pain.^[4,5]^

Many patients with LE (lateral epicondylitis) can be treated without surgery. Many non-surgical treatments for LE, such as physical therapy, drugs, and injection, have been investigated; however, which method is more effective, remains controversial.^[6,7]^

Acupuncture is the traditional therapy of inserting a needle into the skin to stimulate the muscles, nerves, and other connective tissues,^[8]^ providing treatment effects on pain, immune disorders, metabolic diseases, etc. It has been widely used for relieving pain associated with musculoskeletal disorders, and several studies show that patients with LE were satisfied with acupuncture treatment in terms of pain relief and improved dysfunction of the forearm, without adverse events.^[6,9–11]^ As for the analgesic mechanism of acupuncture, research suggests that neural signaling and mechanical activation mainly concerned with opioid peptides and adenosine and central nervous system reactions are likely.^[8,12]^

A review published in 2020^[13]^ assessed the effectiveness of acupuncture for LE. However this review included studies only comparing the efficacy of acupuncture with other therapies (only sham acupuncture, drugs, and steroid injection) but excluded studies comparing acupuncture plus conventional treatment with conventional treatment alone. Furthermore, more recent studies, which were not included in published reviews were searched preliminarily.

We will conduct a systematic review and meta-analysis of randomized controlled trials comparing acupuncture with conventional treatment or acupuncture plus conventional treatment with conventional treatment alone.

## 2 Object

The purpose of this review is to establish comprehensive evidence of the efficacy of acupuncture for LE.

## 3 Methods

### 3.1 Study registration

The study has been registered at PROSPERO (CRD42020186824; https://www.crd.york.ac.uk/prospero/) and follows the preferred reporting items for systematic reviews and meta-analyses protocols 2015 statement.^[14]^

### 3.2 Ethical review

Ethical approval is not necessary because individual patient data will be not included.

### 3.3 Types of studies

To evaluate the effect of acupuncture on lateral epicondylitis, only randomized controlled trials comparing acupuncture with conventional treatment or acupuncture plus conventional treatment with conventional treatment alone for LE will be included. Non- randomized controlled trials, quasi- randomized controlled trials, case reports, reviews, observational studies, and non-clinical studies will be excluded.

### 3.4 Types of patients

Patients diagnosed with LE will be included. There are no limitations in terms of age, sex, race, or intensity or duration of symptoms.

### 3.5 Types of intervention and comparisons

Any type of acupuncture involving insertion of a needle, such as acupuncture, electroacupuncture, and fire acupuncture, will be included as the intervention, regardless of the needle type; site, number, or duration of needling; total treatment period; or frequency of treatment session. Non-dry needling acupuncture, such as pharmacoacupuncture or bee venom, or non-inserting type acupuncture, such as laser acupuncture will be excluded. In the control group, conventional treatments for LE such as physical therapy, anti-inflammatory drugs, corticosteroid injection and extracorporeal shock wave therapy, will be included.

We will include studies using acupuncture or acupuncture with conventional treatment in the experimental group and exclude studies using acupuncture in both experimental and control groups to determine which type of acupuncture is more effective.

In summary, the following types of intervention will be included:

1. Acupuncture versus conventional treatment.
2. Acupuncture plus conventional treatment versus conventional treatment alone.

### 3.6 Data sources

#### 3.6.1 Electronic search

We will search 9 databases from their inception to May 2020, without language or publication status restrictions, including 3 English databases (MEDLINE, Embase, the Cochrane Central Register of Controlled Trials), 5 Korean databases (Korean Medical Database, KoreaMed, Korean Studies Information Service System, Research Information Service System, Oriental Medicine Advanced Searching Integrated System), 1 Chinese database (China Knowledge Network Database).

The following search terms will be used: “acupuncture”, “electroacupuncture”, “fire acupuncture”, “lateral epicondylitis”, “tennis elbow”, “lateral epicondylalgia”, “lateral elbow pain”, “randomized controlled trials”. On Korean and Chinese databases, the aforementioned terms will be translated into Korean and Chinese, respectively, for search. Table 1 shows the search strategy for MEDLINE.

**Table 1:**
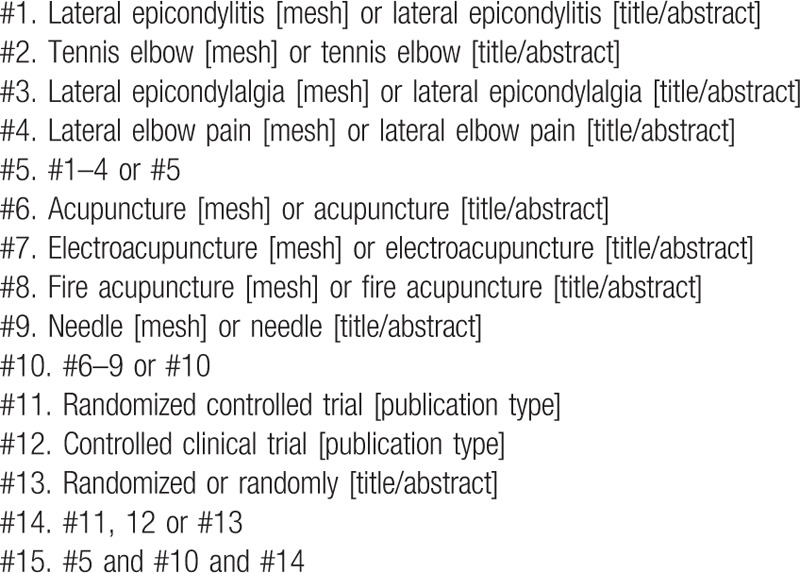
Search strategy for MEDLINE.

### 3.7 Outcome measures

#### 3.7.1 Primary outcomes

The primary outcome will be the pain intensity on a pain scale, such as the visual analogue scale.

#### 3.7.2 Secondary outcomes

Secondary outcomes will include:

1. Grip strength as an indicator of functional recovery
2. Total effective rate
3. Incidence of adverse events

## 4 Data collection and analysis

### 4.1 Selection of studies

**Figure 1:**
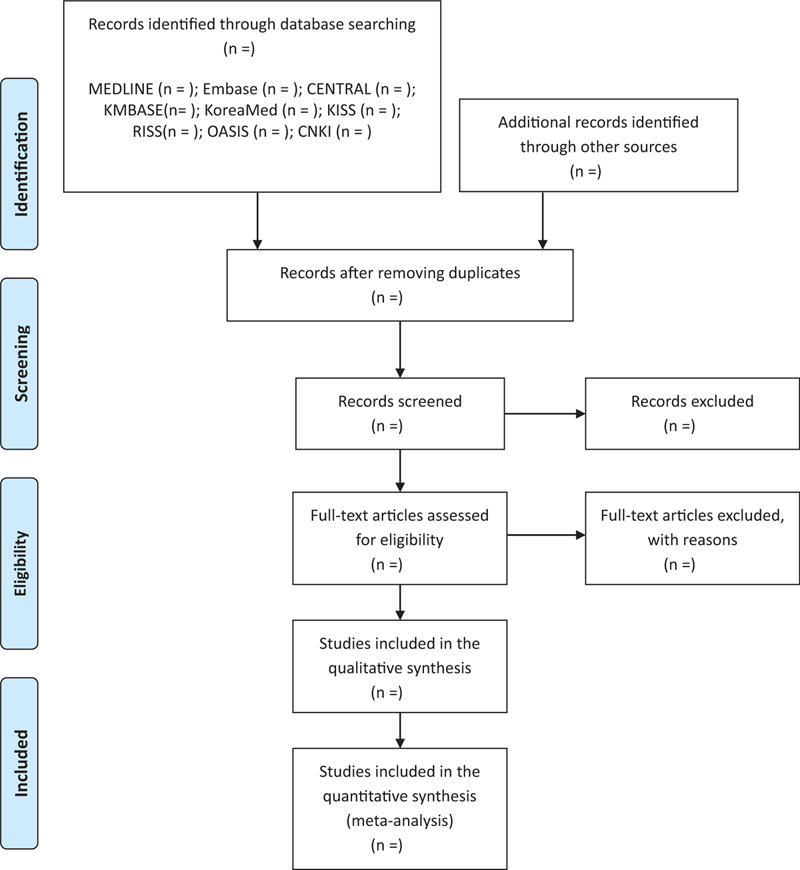
PRISMA flow diagram. CENTRAL = Cochrane Central Register of Controlled Trials, CNKI = China National Knowledge Infrastructure, KISS = Korean Studies Information Service System, KMBASE = Korean Medical Database, OASIS = oriental medicine advanced searching integrated system, PRISMA = preferred reporting items for systematic reviews and meta-analyses, RISS = research information service system.

Two reviewers (H-NK and BG) will screen the title and abstract of all searched articles to exclude duplicated studies and unsuitable articles independently. Subsequently, remaining full texts will be assessed for eligibility according to the inclusion criteria. Any disagreement between 2 reviewers regarding the eligibility of articles will be resolved through a discussion, and if unresolved, a third reviewer (S-SN) will make the final decision. The process of selection will be shown in the preferred reporting items for systematic reviews and meta-analyses flow chart (Fig. 1).

### 4.2 Data extraction and management

Two reviewers (H-NK and BG) will independently extract data from the included studies and fill the predefined data form. Extracted data will include the general information (title, authors, source and year of publication, and country), study characteristics (study design, sample size, randomization method, allocation concealment, and blinding method), participant characteristics (inclusion criteria, age, sex, race, and intensity and duration of symptoms), interventions (types of acupuncture and conventional treatment, details of the treatment method, and frequency and duration of treatment), and outcomes (primary and secondary outcomes). All collected data will be cross-checked and entered into review manager software for the analysis. Any disagreement between 2 reviewers will be resolved through discussions, and if unresolved, a third reviewer (S-SN) will make the final decision.

### 4.3 Assessment of the reporting quality and risk of bias

The risk of bias for each included study will be assessed using the risk-of-bias assessment tool in the Cochrane Handbook. Six domains will be assessed by 2 reviewers (H-NK and BG) independently, including the random sequence generation; allocation concealment; blinding of participants and personnel; blinding of outcome assessment; incomplete outcome data; selective outcome reporting; and other biases, if necessary. For each domain, the included study is assessed to have a “low”, “high” or “unclear” risk of bias. Any disagreement between 2 reviewers will be resolved through discussions, and if unresolved, a third reviewer (S-SN) will make the final decision.

### 4.4 Measures of a treatment effect

Continuous data will be expressed as mean and 95% confidence interval, and dichotomous data will be expressed as risk ratio and 95% confidence interval.

### 4.5 Management of missing data

For missing information, if any, we will contact the corresponding authors. If there is no response, the study will be excluded.

### 4.6 Assessment of reporting biases

We will conduct the assessment of reporting biases, if the number of studies included in the analysis is sufficient, that is, more than 10, using a funnel plot.

### 4.7 Assessment of heterogeneity

Heterogeneity will be assessed with the X^2^ test (A *P*-value < .1 will be considered significant.) and I^2^ value (I^2^ ≥ 50% will be considered to indicate substantial heterogeneity.). For substantial heterogeneity, the reason causing heterogeneity will be additionally studied with a subgroup analysis. If there is substantial heterogeneity, we will conduct a subgroup analysis for the following: intensity of symptoms (pain and grip strength), types of acupuncture therapy and types of conventional treatment (drug, injection, and other therapies).

### 4.8 Data synthesis and grading of quality of evidence

We will perform a meta-analysis, if possible, using the Review Manager 5.3 (Copenhagen; the Nordic Cochrane Center, the Cochrane Collaboration, 2014). To calculate the effect size, fixed- and random-effects models will be used if the result of statistical heterogeneity represents a *P*-value > .1 or, <.1, respectively. If there is substantial heterogeneity, a meta-analysis cannot be performed. In that case, we will provide a narrative and qualitative summary of the included studies. The grading of recommendations assessment, development and evaluation^[15]^ approach is used to assess the certainty of evidence and grade the strength of recommendation.

### 4.9 Sensitivity analysis

A sensitivity analysis will be conducted to confirm the robustness of the results when possible, according to the methodological quality, sample size, and missing data. Further, an analysis will be re-conducted after excluding low-quality studies.

## 5 Discussion

Lateral epicondylitis is a common cause of elbow joint pain, and 16% of the patients complain of work restriction because of the pain and functional disability of the forearm.^[1]^

Despite conservative treatment for 6 to 12 months, if symptoms persist, surgery is considered for approximately 8% of patients. However, conservative treatment is considered primary and is successful in more than 90% of cases. Conservative treatment, includes resting, bracing, physical therapy, anti-inflammatory drugs, corticosteroid injections, botulinum toxin A injections, platelet-rich plasma injections, prolotherapy, and extracorporeal shock wave therapy.^[3]^ Many studies on the treatment for LE have been conducted, but there is still no consensus on which of the many conservative treatments is the most effective.^[6,7,16]^

Acupuncture is commonly used for acute and chronic pain with musculoskeletal disorders, and the effect of acupuncture on pain has been demonstrated. Many studies have proven the efficacy of acupuncture for pain, including lateral epicondylitis,^[10,17,18]^ and patients with LE were satisfied with acupuncture treatment, in terms of pain relief and improved dysfunction of the forearm, without adverse events.^[6,10]^ Therefore acupuncture can be considered as a conservative treatment for LE.

The pain relief mechanism of acupuncture remains partly unclear. Many studies have suggested the gate control theory and release of neurotransmitters or endogenous opioids as the hypothesis.^[17,19]^ Additionally, several studies investigating how acupuncture is therapeutic for tendinopathy have suggested that acupuncture may increase blood flow and collagen proliferation to help regenerate the tendon. Recently, LE has been regarded as a degenerative condition from repetitive damage rather than an inflammatory condition. The effect of acupuncture on recovery from the damaged tendon provides a basis for performing acupuncture to treat LE as an alternative treatment.^[20,21]^

Although the mechanism is not clear yet, many patients have sought to acupuncture therapy because of its analgesic effect and safety in clinical, and it is also affirmed as an alternative treatment by the National Institutes of Health (NIH) when other conventional treatment was not effective than expected.^[8,17,19,22]^

In a previous review,^[13]^ the effect of acupuncture treatment on LE was revealed, but there were some limitations. Furthermore a recent study has also been performed. Therefore, we will conduct this review comprehensively to establish a literary basis for acupuncture treatment for LE including the latest studies. We will carry out this systematic review and meta-analysis to evaluate the efficacy of acupuncture for LE in terms of pain relief and improvement in function and safety. This review can help clinicians and patients set the direction of treatment, and confidently practice acupuncture as the alternative treatment for LE.

## Author contributions

**Conceptualization:** Sang-Soo Nam.

**Investigation:** Bonhyuk Goo, Ha-Na Kim.

**Methodology:** Bonhyuk Goo.

**Project administration:** Ha-Na Kim.

**Writing-original draft:** Ha-Na Kim.

**Writing-reviewing&editing:** Bonhyuk Goo, Sang-Soo Nam.

**Supervision:** Sang-Soo Nam.

**Funding acquisition:** Bonhyuk Goo.
